# Leadership, management and teamwork learning through an extra-curricular project for medical students: descriptive study

**DOI:** 10.1590/1516-3180.2014.1325685

**Published:** 2014-07-29

**Authors:** Maria Lucia da Silva Germano Jorge, Izabel Cristina Meister Coelho, Mariana Martins Paraizo, Ester Fogel Paciornik

**Affiliations:** I MSc, PhD. Researcher, Department of Research and Post-graduation, Faculdades Pequeno Príncipe, Curitiba, Paraná, Brazil.; II MD, Msc, PhD. Researcher, Department of Research and Post-graduation, Faculdades Pequeno Príncipe, Curitiba, Paraná, Brazil; III MSc. Student, Instituto de Pesquisa Pelé Pequeno Príncipe, Curitiba, Paraná, Brazil

**Keywords:** Teaching, Education, medical, undergraduate, Leadership, Education, medical, Curriculum, Ensino, Educação de graduação em medicina, Liderança, Educação médica, Currículo

## Abstract

**CONTEXT AND OBJECTIVE:**

Professionalism in medicine requires preparation for the globalized world. Our objective was to describe a project that introduces medical students to the community, hospital and laboratory activities, thereby allowing them to gain experience in people management, leadership and teamwork.

**DESIGN AND SETTING:**

Descriptive study of the process applied at a philanthropic medical school in Curitiba, Paraná.

**METHOD:**

Inclusion of management and leadership practices as part of the medical degree program.

**RESULTS:**

The study groups consisted of fifteen students. After six months, any of the participants could be elected as a subcoordinator, with responsibility for managing tasks and representing the team in hospital departments and the community. The activities required increasing levels of responsibility. In medical schools, students' involvement in practical activities is often limited to observation. They are not required to take responsibilities or to interact with other students and stakeholders. However, they will become accountable, which thus has an adverse effect on all involved. The learning space described here aims to fill this gap by bringing students closer to the daily lives and experiences of healthcare professionals.

**CONCLUSION:**

Being a physician requires not only management and leadership, but also transferrable competencies, communication and critical thinking. These attributes can be acquired through experience of teamwork, under qualified supervision from teaching staff. Students are thus expected to develop skills to deal with and resolve conflicts, learn to share leadership, prepare others to help and replace them, adopt an approach based on mutual responsibility and discuss their performance.

## Abstract (pt)

**CONTEXTO E OBJETIVO:**

Profissionalismo na medicina requer formação para o mundo globalizado. Nosso objetivo é descrever um projeto que introduz os estudantes de medicina em atividades laboratoriais, comunitárias e hospitalares, oferecendo experiência em gestão de pessoas, liderança e trabalho em equipe.

**TIPO DE ESTUDO E LOCAL:**

Estudo descritivo do processo aplicado a uma escola medica filantrópica de Curitiba, Paraná.

**MÉTODO:**

Inclusão de práticas de gestão e liderança como parte do programa de graduação médica.

**RESULTADOS:**

Os grupos de trabalho eram constituídos por 15 estudantes. Após seis meses de participação, o estudante podia ser eleito como subcoordenador, responsável por gerenciar tarefas e representar a equipe junto aos setores do hospital e na comunidade. Na faculdade de medicina, frequentemente, o envolvimento dos alunos em atividades práticas restringe-se à observação, não existem demandas sobre a sua responsabilidade ou sua interação com outros alunos e atores. Entretanto, serão cobrados por isso, resultando em efeito adverso sobre todos os envolvidos. O espaço de aprendizagem aqui descrito visa preencher essa lacuna, trazendo os alunos para mais perto do cotidiano e das experiências dos profissionais de saúde.

**CONCLUSÃO:**

Ser médico exige, ao lado de gestão e liderança, competências transferíveis, comunicação e pensamento crítico. Atributos adquiridos pela experiência do trabalho em equipe sob supervisão qualificada do corpo docente, quando os estudantes devem desenvolver habilidades para lidar e resolver conflitos, aprender a compartilhar a liderança, preparar outras pessoas para ajudar e substituir, adotar uma abordagem baseada na mútua responsabilidade e na análise de desempenho.

## INTRODUCTION

In the globalized information age, cultural miscegenation and the ease with which people can travel have given rise to new values and needs. Linked to these changes is the quality of people's health, which is one of the main focuses of attention. Healthcare systems, both public and private, seek to adapt to these changes by dedicating particular attention to disease prevention, constant technological development and scientific discoveries. However, consolidation of healthcare, which is universally sought, is linked to the availability of qualified healthcare professionals.^1^^,^ 2

One important point of reference among healthcare professionals is the physician, whose training must include far more than clinical reasoning and technical skills. Communication skills, responsibility, altruism, humanistic attitudes and other competencies relating to emotions, as well as an understanding of the ethical and legal aspects of medicine and training in dealing with community interests are essential.^3^^,^ 4

Producing professionals with these qualifications requires constant improvements and the development of learning processes that include these competencies. In this context, the Flexner report stressed the need for scientific research to be included in medical training and the importance of teaching scientific principles and methods, evidence-based medicine, critical thinking and the solution of practical problems.^5^

Nonetheless, after many studies on the application of scientific research as a teaching method and learning process, undergraduate teaching and research continue along separate paths in many institutions.^6^ While the two areas often share the same physical space in teaching institutions, and in some cases a strategy is adopted in which undergraduate students are introduced to and apply scientific methods, this is done merely to encourage them to go on to postgraduate studies.^7^^,^ 8

To change this situation, the traditional curriculum needs to be reorganized by including technically oriented programs that offer scientific experiences integrating basic, applied and vocational training. 

## OBJECTIVE

This paper sought to describe an optional curricular activity that was designed to introduce students to the hospital community and provide guidance in practical and management activities in a quality control laboratory.

## METHODS

This project was introduced in a private, not-for-profit, philanthropic medical school with a traditional curriculum covering basic scientific subjects and vocational clinical training separately, mainly through guided hospital-based teaching. The findings from the study were applied directly in the community where the study was carried out.

The team, consisting of a member of the teaching staff and students, provided services for the university hospital associated with the institution, and their duties included carrying out legally required quality control tests to control nosocomial infections according to the demand from the hospital's infectious disease control committee (IDCC). The teaching, administrative and laboratory activities were carried out in the university laboratories. The study covers the period 2003 to 2010. 

## RESULTS

### Project organization

The microbiological analysis involved testing the water throughout the hospital as well as products from the food and nutrition department and all pasteurized material in the human milk bank. The activities were divided between three teams of medical students in their 5th to 10th semesters. The selection process for the fifteen students included a test on biological safety, quality control, microbiological diagnostics and infectious diseases followed by examination of candidates' résumés and an interview. Once approved, all the students started by learning how to wash and sterilize material, prepare culture media and carry out maintenance and quality control on equipment (refrigerator, freezer, autoclave and microwave oven).

The next stage involved collecting samples and carrying out laboratory tests. Following this, work protocols were prepared and standard operating procedures updated. Finally, the team members analyzed and recorded the data and discussed their results with their peers and various hospital departments. At this point, sub-coordinators could be elected. The tasks were organized and distributed every month according to the needs of the different hospital departments and students' learning objectives and academic activities. As students' aptitudes for certain tasks became apparent, they were gradually assigned tasks that were more technically complex and demanded greater responsibility. 

The last stage in the training required students to coordinate the team working with human milk. Although this activity was technically easier and less demanding than the previous ones, it involved a high degree of responsibility because the material was released directly for consumption. 

Each student spent an average of ten hours on project activities every week. One constant source of concern was the need to ensure that students' academic performance was not adversely affected. During the study period, project productivity increased significantly: a total of 1380 analyses were carried out in 2003, while in 2009 the corresponding figure was 5100, or 420 samples per month on average.

### Management training program

The main reason for organizing the project in this way was to prepare students for team activities and roles requiring them to exercise leadership. Each group consisted of a subcoordinator and four or five students in different semesters. Every six months, one of the eligible candidates was elected subcoordinator by the team members. Reelection was not allowed.

Four-hour weekly meetings were held to analyze and discuss questions relating to leadership, management, administrative, technical and educational issues, interpersonal relationships, safety procedures, analysis of results, analytical methods and any difficulties that had occurred in the previous week. Students were actively encouraged to correlate their experiences in medical school with their project activities, to examine the data scientifically, to compare the results with the literature and to take part in scientific events. 

Minutes of the meetings were recorded and made fully available to students. The information in these served as a database that could be used for decision-making, further studies and assessment of the team's activities. The students who took part in this project were supervised and worked with the mothers who were contributing to or using the milk bank and with staff from the various departments. 

They spent time in the quality control laboratory, learning "hands on" about the legal requirements for controlling hospital infection, and carried out different tasks, from basic technical work to personnel management. Over the course of the study period, one student was asked to leave because of problems adapting to the project, and two left for personal reasons. By 2010, 56 students had taken part in the project, and 26 of these had completed their medical studies.

## DISCUSSION

This paper describes an enriching experience involving a teaching method and learning space that were used to introduce students to a simple working environment that can easily be set up. The study not only provided an opportunity for students to take part in activities in a hospital setting and in the satellite communities, but also met the needs of the IDCC and the community for the services that the students provided.

The project also stimulated development of leadership and management competencies and skills, teamwork and interpersonal relations among the students, and accountability for the results of their work and the consequences of these results. A learning process like this needs to result in a product for students, so that they can appropriate and share knowledge and recognize other perspectives and possibilities for conceiving, perceiving and explaining reality. 

Learning spaces are constructed by articulating learning and work processes and allowing students to participate in the formulation of alternative procedures and interventions.^9^^-^ 11 In a globalized world, it is of particularly importance to produce students who can occupy leadership and management positions and work in teams and, above all, who are ready to take up political and administrative positions in the academic world.^12^^,^ 13

Students in medical school take part in various activities alongside multiprofessional teams, fellow students and patients, but their roles in these are merely observational. No demands are made on them in terms of a requirement for them either to take responsibility for their actions or to interact with fellow students and other stakeholders. Furthermore, students are in general only required to be accountable for the results of their work and the consequences of these results during the final years of their course, a situation that naturally has an adverse effect on all those involved.^10^

The learning space described here has brought medical school students closer to the daily lives and experiences of healthcare professionals, has required them to be accountable for their professional activities and has stimulated communication and results from the first semesters of their course.^14^

According to de Souza et al., promoting collaboration between staff who teach basic, applied and vocational subjects and non-teaching healthcare professionals represents a major challenge. It is therefore difficult, in a project that combines basic and applied subjects with vocational practice, to ensure that healthcare measures are effective for the community. However, if teamwork is undertaken based on the needs and work routines of teaching and non-teaching health professionals and lecturers in basic subjects, all those involved will be able to work as they normally do, but together, thereby motivating student-oriented learning.^9^

Despite the fundamental importance of scientific research in producing critical, reflective professionals, it is considered by medical students to have little relationship to patients and individuals. A qualitative survey of undergraduate medical students' perceptions of research found that more than a quarter (60/317) of those who took part made negative comments about it.^12^ This can be explained by the difficulty that medical students have in understanding the concepts involved in scientific research, starting with the professor/researcher situation. Teaching staff do not normally have the necessary background to produce students equipped with a knowledge of scientific methodology. 

However, in comparing medical students from two courses with different curricula, Pruskil et al. found that individuals were more confident about their own scientific competencies when they had had more research opportunities. Many medical students identified research as something remote, a misconception that can be explained by the lack of research activities in medical programs.^10^

Putting students into a position in which they need to apply their acquired knowledge to day-to-day activities, while making reference to scientific articles and comparing results, favors development of critical thinking. Similarly, in a statement about the purpose of scenarios in physicians' development, Blank proposed a new paradigm for an integrated approach that would allow physicians to intervene in a health-disease process within the overall context of the process, while balancing technical issues with social and behavioral issues.^15^

The aim of any medical school is to prepare students to practice medicine in an ethical, competent and socially responsible manner. It is very important to produce professionals who have an overall vision and aptitudes that span a variety of processes rather than just knowledge of medical procedures. Likewise, in addition to being able to solve clinical problems, students must work as researchers to enable them to be leaders in the healthcare community.^16^ To achieve this goal, a curriculum supported by educational programs that help students develop critical analysis, resolve problems and manage people is fundamental.

## CONCLUSION

Nowadays, being a physician requires not only an understanding of management and leadership but also transferrable competencies, such as communication, teamwork, time management and critical thinking. These attributes can be acquired by exposure to teamwork, thus giving students the opportunity to use the knowledge that they have acquired, in real situations in which they are in charge under the supervision of members of the teaching staff. In this way, students can acquire the skills to deal with and solve conflicts, learn to share leadership, prepare others to help and replace them, approach their work with an attitude of mutual responsibility and hold discussions on detailed aspects of their performance. The model described here is notable for its potential, and consideration should be given to its inclusion in curricula in medical schools.
